# Effect of Bovine Colostrum Dietary Supplementation on Rabbit Meat Quality

**DOI:** 10.3390/foods11213433

**Published:** 2022-10-29

**Authors:** Marta Castrica, Laura Menchetti, Stella Agradi, Giulio Curone, Daniele Vigo, Grazia Pastorelli, Alessia Di Giancamillo, Silvia Clotilde Modina, Federica Riva, Valentina Serra, Dino Miraglia, Egon Andoni, Gabriele Brecchia, Claudia Maria Balzaretti

**Affiliations:** 1 Department of Veterinary Medicine and Animal Sciences, University of Milan, Via dell’Università 6, 26900 Lodi, Italy; 2 School of Biosciences and Veterinary Medicine, University of Camerino, Via Gentile III da Varano, 62032 Camerino, Italy; 3 Department of Biomedical Sciences for Health, University of Milan, Via Mangiagalli 31, 20133 Milan, Italy; 4 Department of Veterinary Medicine, University of Perugia, Via San Costanzo 4, 06126 Perugia, Italy; 5 Veterinary Faculty of Tirana, Department of Public Health, Agricultural University of Tirana, Rr Pajsi Vodica, Koder-Kamez, 1029 Tirana, Albania

**Keywords:** rabbit, meat microbial profile, TBARS, functional food, bioactive compounds

## Abstract

Bovine colostrum (BC) is rich in nutrients, antimicrobial, and antioxidant factors; for these reasons, it has been used as supplement in animal nutrition. However, its possible effects on meat quality have not been studied yet. Thirty-nine New Zealand White rabbits (*n* = 13/group) were assigned to three groups and fed until slaughter with a commercial standard diet, control group (C), and C supplemented with 2.5% and 5% (*w/w*) of BC (BC-2.5 and BC-5 groups, respectively). After slaughtering, the effect of dietary supplementation on microbiological and chemical characteristics of the rabbit loins was evaluated at 48 h postmortem (D0) and after 3 (D3) and 8 (D8) days of refrigerated storage. Results showed no difference in the microbiological parameters. In the supplemented groups, TBARS and TVBN values were lower and higher than in the C group, respectively (*p* < 0.01), and their fatty-acid profile was increased in SFA and decreased in MUFA (*p* < 0.05). In conclusion, research must continue to examine in depth the possible effects of BC byproduct reuse in animal nutrition on meat quality (e.g., antioxidant power, and physical and sensory characteristics).

## 1. Introduction

Growing attention is now being given to nutraceuticals and functional foods, which have been widely used and studied for their beneficial effect on human and animal health and welfare [1,2]. Nutraceutical substances are represented by foods or their parts that carry out physiological and biological activities and that can determine the prevention or treatment of specific diseases or disorders [3,4]. Functional foods are those which, beyond their basic nutritional properties, can positively influence one or more physiological functions [5,6,7].

A fundamental prerogative of these foods is also in helping to preserve or improve the state of health of individuals [8,9]. In this context, colostrum can be considered an excellent nutraceutical. Colostrum is a nutrient-rich fluid secreted by female mammals [10], and it plays an important role as an immune booster in postnatal health [11]. Specifically, bovine colostrum (BC) is the early milk produced in the first 4 days of lactation [12]. BC is a wealthy resource of biologically active compounds, such as antimicrobials, antioxidants, essential nutrients, and immune-regulating factors [13]. It is very complex to provide a standard composition and physical characteristics of BC because it is affected by several factors such as individual breeds, number of calves, length of dry period and length of period elapsed since parturition, animal health, farm management, and feeding [14,15].

BC’s functional attributes have been known for thousands of years, and its importance was initially described in ancient Ayurveda medicine [16]. Many of the now-known effects of BC supplementation have been evaluated through experimental studies using different animal models such as mice [17], rats [18], rabbits [19], lambs [20], poultry [21], and calves [22]. In 1799, Hufeland was the first to describe a straight relationship between the intake of bovine colostrum by calves and their rapid and healthy growth, thus triggering scientific interest in this compound that has led, over time, to in-depth studies of its properties and possible uses [23]. In recent years, BC has been used in the prevention and treatment of different human diseases [24,25,26,27,28,29,30,31,32,33,34,35]. The immune factors present in BC make its prophylactic and therapeutic use in the prevention and treatment of bacterial and viral infections effective. Thanks to the presence of 20 specific antibodies, the BC is capable of exerting an antimicrobial and antiviral action against *E. coli*, *Salmonella*, rotavirus, *Candida*, *Streptococcus*, *Staphylococcus*, *Cryptosporidium*, and *H. pylori* [36].

In the last decades, several nutraceuticals, including BC, have also been used in the zootechnical field to increase the immune status [37,38,39], to reduce the incidence of some pathologies and the mortality [40,41,42] and to improve the productive performance of various livestock animals among which pigs [43], broilers [44], lambs [20,45], and calves [46].

Nonetheless, studies in livestock animals, especially rabbits, are limited mainly with regard to possible positive effects of BC dietary supplementation in improving meat quality in terms of microbiological, chemical, and sensory profile.

The rabbit is a very interesting species because it is bred for different purposes: for research, as a pet, and as a livestock animal [47,48,49,50]. Rabbits are intensively bred in the world, especially in China and Europe, particularly in the countries of the Mediterranean area, including Italy [51,52]. It represents the fourth livestock sector in economic importance after cattle, pigs, and chickens [53]. Rabbit meat is appreciated by consumers for its high nutritional value and dietetic and sensory proprieties. This type of meat shows high protein levels, very low fat concentrations (particularly cholesterol), and a high percentage of unsaturated fatty acids [54,55]. In this context, finding nutraceutical products to be added to the standard animal feed, which can potentially amplify the healthy value of animal origin product, is a strategy worth aiming for. Additionally, byproducts rich in bioactive substances are now being highly explored within the agro-food chain because they represent a sustainable strategy for enhancing food quality through the reuse of byproducts otherwise destined for disposal [6,56,57]. By contrast, the quality of animal productions in relation to the dietary intake of colostrum has not yet been investigated, although its health properties have been well recognized. Moreover, given that most healthy cows produce far more colostrum than the calf needs and that colostrum in surplus is not marketable, its reuse in animal husbandry is an excellent circular economy strategy. For these reasons, this study aimed to determine the effect of dietary supplementation with two different concentrations of BC, 2.5% and 5%, on the microbiological and chemical quality of rabbit meat.

## 2. Materials and Methods

### 2.1. Animals and Diets

The experiment was carried out at the Department of Agricultural, Food, and Environmental Science of the University of Perugia’s experimental farm and was conducted in accordance with the Legislative Decree No. 146, implementing Directive 98/58/EC of 20 July 1998 concerning the protection of animals kept for farming purposes. Post weaning, 39 New Zealand White (NZW) rabbits (*n* = 13/group) were randomly divided into three groups according to the type of diet administered; the control group (C) was fed with a commercial standard diet (CSD), while the other two groups received a CSD supplemented with 2.5% and 5% bovine colostrum, BC-2.5 and BC-5 groups, respectively (Table 1). At the end of the growth trial (91 days of age), all animals were slaughtered.

### 2.2. Meat Analyses

After slaughtering, the two *Longissimus dorsi* muscles (*n* = 26/group) of each rabbit belonging to the three groups (C, BC-2.5, and BC-5), whose subcutaneous fat was removed, were separated, and then were randomly packed in an oxygen-permeable package consisting of expanded polystyrene tray covered with PVC film, where the characteristics of PVC film with regard to O_2_ and H_2_O vapor permeability are given in Schlimme and Rooney [58]. After packing, all the samples were transported to the Food Inspection Laboratory at Department of Veterinary Medicine and Animal Sciences, University of Milano (Italy), under refrigerated conditions and stored in the dark at 4 ± 1 °C until the analyses were performed.

The rabbit loin quality was evaluated at 48 h postmortem (day 0; D0) and after 3 (D3) and 8 (D8) days of storage. For each group and for each sampling time, 13 left loins were tested for physicochemical parameters and 13 right loins were tested for microbiological parameters.

#### 2.2.1. Chemical Composition

At D0, meat samples were evaluated for moisture, fat, protein, and ash according to AOAC (Association of Analytical Chemists, 2000) using methods 950.46, 960.30, 992.15, and 923.03, respectively. The fatty-acid profile in meat was evaluated according to Chiesa et al. [59]. Briefly, the fatty-acid analysis was performed on a GC (TRACE GC Ultra, Thermo Fisher Scientific, Rodano, Italy) equipped with an automatic sampler (AI 1300, Thermo Fisher Scientific, Rodano, Italy) and FID detector. An RT-2560 fused silica capillary column (Restek, Milan, Italy) was used with a programmed temperature from 80 °C to 180 °C at 3 °C·min^−1^, and then 180 °C to 250 °C at 2.5 °C·min^−1^, which was held for 10 min. The carrier gas was helium at 1.0 mL·min^−1^ with an inlet pressure of 16.9 psi. A quantitative procedure was used where 1 mL of internal standard (1 mg·mL^−1^ 23:0 methyl ester; N-23-M; Nu-Chek Prep Inc., Elysian, MN, USA) was added before methylation. The fatty acid methyl ester (FAME) contents were quantified (by weight) as a percentage (%) of the total FAMEs. All analyses were performed in duplicate. Moreover, indices of atherogenicity (AI) and thrombogenicity (TI) were calculated as suggested by Ulbricht and Southgate [60].

#### 2.2.2. Microbiological Analysis

For counting *Staphylococcus aureus*, *Enterobacteriaceae*, *Escherichia coli* and total coliforms, for the analysis times selected, 10 g of sample was aseptically placed in a stomacher bag with 90 mL of sterile buffer peptone water (BPW; Oxoid, Basingstoke, UK), and then mixed together for 2 min. After this step, 1:10 serial dilutions of the samples were prepared with sterile BPW. A 1 mL aliquot from each diluted sample was plated in duplicate on 3M Petrifilm (3M, St. Paul, MN, USA). The 3M Petrifilm consists of a sample-ready culture medium system which contains a cold-water-soluble gelling agent. Specifically, *S. aureus* petrifilm has a chromogenically modified Baird-Parker medium, *Enterobacteriaceae* contains modified Violet Red Bile Glucose (VRBG) nutrients, and *E. coli* and total coliforms plate are composed of modified Violet Red Bile (VRB) nutrients and an indicator of glucuronidase activity, 5-bromo-4-chloro-3-indolyl-D-glucuronide (BCIG). With regard to *Enterobacteriaceae*, *E. coli* and total coliforms, also contain a tetrazolium indicator that facilitates colony enumeration. The results were expressed as log CFU/g, and, when no colonies were detected, the value indicated was <1 log CFU/g (spread plating of 1 mL from the sample).

#### 2.2.3. Total Volatile Basic Nitrogen and Lipid Oxidation Analysis

The TVBN and lipid oxidation (TBARS) assays were performed as reported by Castrica et al. [6]. TVBN was determined using a VELP Marka model UDK 139 apparatus (Velp Scientifica, Usmate, Milan, Italy). In brief, 10 g of sample was alkalized with 2 g of magnesium oxide, and then distilled and titrated with 0.01 N HCl. The results were expressed as mg/100 g of sample. Regarding TBARS, 10 g of the sample was homogenized with 50 mL of distilled water, and the resulting mixture was transferred to a Kjeldahl flask by washing with another 47.5 mL of distilled water. Next, 2.5 mL of HCl (4 N) solution was added to raise the pH to 1.5. Then, an antifoaming agent and some saddle stones were introduced to prevent bumps. After that, the apparatus was assembled, and the flasks were heated to the maximum heat obtainable on the Kjeldahl distillation apparatus; then, 50 mL of distillate was collected within 10 min of the onset of boiling. After that, 5 mL of distillate were pipetted into a 50 mL stoppered glass tube, and 5 mL of TBA reagent was added; the tube was closed, and the content was mixed and immersed in a boiling water bath for 35 min. At the same time, a blank of distilled water and TBA reagent was prepared and treated as a sample. After warming, the tube was cooled in tap water for 10 min. A part of the content was transferred to a 1 cm cuvette, and the optical density was read against the blank in a spectrophotometer at a wavelength of 538 nm; the optical density value was multiplied by a factor of 7.8, and the results were expressed as mg malondialdehyde (MDA)/kg muscle. All analyses were performed in duplicate.

### 2.3. Statistical Analysis

The distribution of microorganisms in rabbit meat was expressed as microbial count (log CFU/g) and prevalence (percentage of units that are contaminated, i.e., count > 1 log CFU/g) [61]. Moreover, for each group and time, the number and percentage of samples exceeding the microbiological limits were calculated [61]. The following microbiological limits were set: *Staphylococcus aureus*, <4 log CFU/g; *E. coli*, ≤4 log CFU/g; *Enterobacteriaceae*, <4 log CFU/g; total coliforms, ≤4 log CFU/g [62].

Differences between groups in terms of chemical composition, microbial count, TBARS, and TVBN at any time and differences over time within each group were evaluated using the Kruskal-Wallis and Mann-Whitney tests, while chi-square and z-tests were used to analyze the prevalence. Statistical analyses were performed using SPSS Statistics version 25 (IBM, SPSS Inc., Chicago, IL, USA) and GraphPad Prism, version 7.0 (GraphPad Software, San Diego, CA, USA). The level of statistical significance was set at *p* < 0.05.

## 3. Results and Discussion

### 3.1. Chemical Composition and Meat Fatty-Acid Profile

Rabbit meat has a low-fat content, and its lipids are highly unsaturated (60% of total FA); it has a high protein content, its amino acids are of high biological value, and it is low in cholesterol [63,64]. In this study, the lipid content of the meat increased with increasing colostrum dose in the diet, even though the differences from the control were significant only for the BC-5 group (*p* < 0.05) (Table 2). This could be related to the high lipid content in bovine colostrum [14,65].

**Figure 1:**
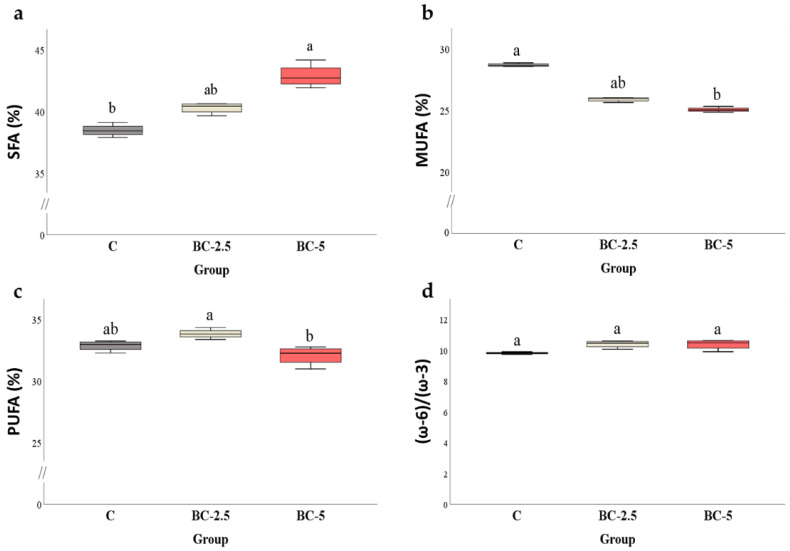
Effects of colostrum inclusion in the diet of rabbits on the content of saturated (SFAs) (**a**), monounsaturated (MUFAs) (**b**), polyunsaturated fatty acids (PUFAs) (**c**), percentage total peak area and ω-6/ω-3 ratio (**d**) of the meat. C = control diet; BC-2.5 = diet supplemented with 2.5% bovine colostrum; BC-5 = diet supplemented with 5% bovine colostrum. *p*-Values are statistically significant at α = 0.05. a, b: Values followed by the same letter do not differ significantly.

From a qualitative point of view, the fatty-acid profile was not influenced by colostrum supplementation, as demonstrated by the same number of FAs (29) identified in all groups (Table 3). Nevertheless, the FA composition of the meat quantitatively resulted in several dose-dependent changes according to the dietary regimen (Table 3). In detail, the dietary supplement with 5% colostrum increased the percentage of saturated fatty acid in the meat by about four percentage points (Figure 1a), mainly due to the increase in stearic (C16:0) and palmitic (C18:0) acids; this was also seen for the latter in the 2.5% group. At the same time, a decrease in MUFA was recorded in BC-5 samples (Figure 1b), substantially due to the reduction in oleic acid (C18:1n9c) (Table 3). As a result, the BC-5 group had greater TI and AI than the control and, for the latter index, also compared to the BC-2.5 group.

Oscillations of some polyunsaturated fatty acids, particularly linolenic acid (C18:3n3) and arachidonic acid (C20:4n6), left the PUFA percentage of experimental groups substantially unchanged compared to the control (Figure 1c). Furthermore, the multiple comparisons showed no difference in the ω-6/ω-3 ratio (Figure 1d), even though the ω-3 sum was significantly lower in BC-5 than in the control group (Table 3).

From a nutritional point of view, dietary supplementation with colostrum had a tendency to modify the FA quality of rabbit meat. The trend became noticeable with 5% BC supplementation, which resulted in a significant increase in SFAs associated with a decrease in MUFAs and the FAs of the ω-3 series. Of the latter, it is interesting to note the decrease in linolenic acid, an essential fatty acid which is very relevant to human health [66,67]. On the contrary, this acid profile can be considered technologically better, making the fat less susceptible to oxidative phenomena [68,69]. In this respect, the MDA produced in the colostrum samples remained unchanged during storage, such that, at the last observation, the values were significantly lower than control, indicating greater stability of the meat during storage (Table 4).

However, it must be emphasized that the antioxidant properties of colostrum may have played a crucial role in delaying oxidative processes in the lipid component.

### 3.2. Meat Microbial Status

**Figure 2:**
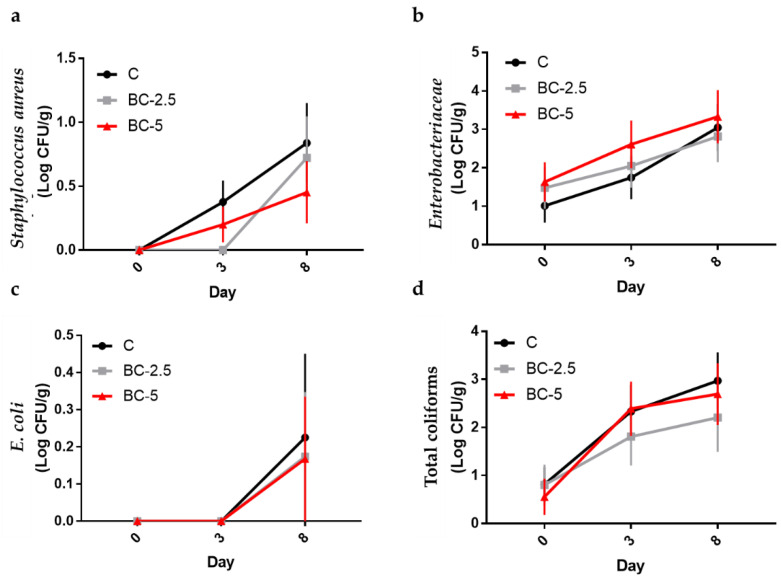
Effects of colostrum inclusion in the diet of rabbits on counts of *Staphylococcus aureus* (**a**), *Enterobacteriaceae* (**b**), *E. coli* (**c**), and total coliforms (**d**) on meat at 48 h postmortem (day 0) and at days 3 and 8 of shelf-life. C = control diet; BC-2.5 = diet supplemented with 2.5% bovine colostrum; BC-5 = diet supplemented with 5% bovine colostrum. The graphs show the mean ± standard error.

As regards the microbial count (Figure 2), there were no differences between groups but an increase over time was found in the C group for *Staphylococcus aureus* (*p* = 0.041), *Enterobacteriaceae* (*p* = 0.019), and total coliforms (*p* = 0.012). In the supplemented groups, significant increases were only found for *Staphylococcus aureus* in BC-2.5 (*p* = 0.013) and for total coliforms in the BC-5 group (*p* = 0.015).

Therefore, it appears that no antimicrobial compounds present in colostrum were transferred to the meat in sufficient quantities to affect microbial populations. These results are in line with findings from previous studies, where supplementation of the animal diet with phytoderivatives did lead to limited hygienic profile advantages [6,70,71]. In our opinion, the strategy of limiting the microbial development of food during shelf-life through animal feeding still needs to be confirmed.

Table S1 shows the number of samples contaminated by each microorganism. A significant difference was found only for *Staphylococcus aureus* at day 3, when the number of positive samples belonging to the BC-2.5 group was lower than that in the control (*p* < 0.05). Thus, from the point of view of percentage contaminated units, the addition of colostrum to the diet also did not appear to be effective in reducing prevalence, except for the BC-2.5 group at D3. Confirmation of this also emerged from the detection of some samples considered unacceptable according to the predetermined limits for *Enterobacteriaceae* and total coliforms, without differences between the groups (Table S2). The level of meat contamination is strongly related to the hygienic level of processing. The detection of certain noncompliant samples for microorganisms, mainly from the intestinal tract already in the first few days of storage (Table S2), can be attributed to incorrect skinning and evisceration procedures.

### 3.3. Meat Oxidative Status and TVBN

The correlation between animal nutrition and the quality characteristics of their meat is well known. In this regard, lipid oxidation plays a fundamental role during product preservation. Delaying this process by supplementing the animals’ diet is a strategy that is being scientifically recognized. Many studies have highlighted the efficacy of plant-derived antioxidant molecules [57,72,73,74], as well as the beneficial properties of colostrum, but the use of colostrum in animal feed to postpone meat oxidation has not yet been investigated. In this study, both dosages of colostrum supplementation positively influenced the oxidative status of the meat. In fact, at the end of the storage period, the supplemented groups showed lower values of TBARS compared to the control (*p* = 0.008) (Table 4). In particular, the MDA produced in the C samples increased significantly over time (*p* = 0.021), reaching double values compared to D0 after 8 days.

This nutraceutical potential results from the different molecules with protective factors against lipid oxidation, as found in the BC and shown in several studies [13]. It should be stressed that lipid oxidation also depends on the amount and composition of fat [75]. The 5% colostrum diet increased the more stable SFAs while decreasing the more unstable MUFAs and ω-3. This may have contributed to slowing down of the oxidative processes, although a higher concentration of ether extract was recorded in this group compared to the control (*p* = 0.01) (Table 2).

An effect of the diet was also observed for TVBN; at the second observation, both treated groups reached significantly higher concentrations than the control, whereas, at the end of the observation period (D8), the values were similar (*p* = 0.146) (Table 4). This is because a significant increase in nitrogen over time (*p* = 0.005) was only found in the control group. TVBN is considered to assess the degradation of the nitrogen component of food during storage. Its trend is conditioned by the activity of tissue and microbial enzymes [76,77]. In this study, the increase in nitrogen over time reflected the increase in microbial populations in the control group (Figure 2). Similar results of TBARS and TVBN were found by the same authors [6] by supplementing the rabbits’ diet with Goji berries. In all cases, the concentrations remained above standards, defined as acceptable by several authors [78,79], throughout the entire storage period.

## 4. Conclusions

The results obtained in this study need further investigation. To date, the lack of literature findings on animal feeds containing colostrum in their formulations and evaluation of meat quality makes it difficult to make comparisons and considerations. Certainly, dietary colostrum supplementation improved the oxidative fatty acids state of rabbits, but it appears that increasing its percentage of supplementation up to 5% in the feed also increased the amount of SFAs to the disadvantage of MUFAs. It is clear that, from a nutritional point of view, such a shift in fatty acids is not desirable. Therefore, this must be further investigated in order to determine the optimal colostrum concentration that allows an antioxidant action on meat, without affecting the health aspect. With regard to the antibacterial activity recognized in colostrum due to its composition, it does not appear that bioactive molecules were transferred from feed to meat. To better understand this issue, it would be interesting to develop in vitro inhibition tests involving different marker microorganisms and different concentrations of colostrum in the animals’ diet. A further interesting focus could be to test colostrum-containing formulations to be used directly on meat before packaging.

## Figures and Tables

**Table 1:** Chemical composition of control (C) and experimental diets supplemented with 2.5% (BC-2.5) and 5% (BC-5) of bovine colostrum (% wet weight).

Ingredient	Basal Diet	Supplemented Diets	
	C	BC-2.5	BC-5
Wheat bran	30.0		
Dehydrated alfalfa meal	42.0		
Barley	9.5		
Sunflower meal	4.5		
Rice bran	4.0		
Soybean meal	4.0		
Calcium carbonate	2.2		
Cane molasses	2.0		
Vitamin–mineral premix	0.4		
Soybean oil	0.4		
Salt	0.3		
Bovine colostrum	-		
**Chemical composition**			
Dry matter	92.34	91.71	91.69
Crude protein	14.82	14.76	15.23
Ether extract	2.79	2.95	3.02
Ash	7.04	7.23	7.62
NDF ^1^	40.00	36.81	35.79
ADF ^2^	27.04	24.92	24.31
ADL ^3^	12.02	10.03	9.11

^1^ NDF: neutral detergent fiber; ^2^ ADF: acid detergent fiber; ^3^ ADL: acid detergent lignin.

**Table 2:** Effect of colostrum inclusion in the diet of rabbits on the chemical composition (g/100 g) of the meat (mean ± standard error; SE). C = control diet; BC-2.5 = diet supplemented with 2.5% bovine colostrum; BC-5 = diet supplemented with 5% bovine colostrum.

	Group						*p*-Value
	C		BC-2.5		BC-5		
	Mean	SE	Mean	SE	Mean	SE	
**Moisture (%)**	78.05	0.18	77.65	0.17	77.51	0.02	0.077
**Protein (%)**	20.33	0.11	20.30	0.11	20.14	0.11	0.492
**Lipids (%)**	0.52 b	0.07	0.94 ab	0.06	1.22 a	0.06	**0.010**
**Ash (%)**	1.11	0.02	1.10	0.04	1.13	0.04	0.776

a, b: Values followed by the same letter in each row do not differ significantly (*p* < 0.05, Bonferroni correction). *p*-Values in bold are statistically significant at α = 0.05.

**Table 3:** Effect of colostrum inclusion in the diet of rabbits on the fatty-acid profile of the meat (mean ± standard error; SE). C = control diet; BC-2.5 = diet supplemented with 2.5% bovine colostrum; BC-5 = diet supplemented with 5% bovine colostrum.

	Group						*p*-Value
	C		BC-2.5		BC-5		
	Mean	SE	Mean	SE	Mean	SE	
C10:0	0.19 a	0.01	0.12 ab	0.01	0.02 b	0.00	**0.007**
C12:0	0.35 a	0.01	0.24 ab	0.01	0.11 b	0.00	**0.007**
C14:0	2.57 b	0.05	2.57 ab	0.09	3.08 a	0.16	**0.024**
C15:0	0.47 b	0.00	0.48 ab	0.01	0.52 a	0.01	**0.031**
C16:0	28.50 b	0.25	28.91 ab	0.20	31.27 a	0.47	**0.021**
C17:0	0.49 b	0.00	0.54 ab	0.00	0.55 a	0.00	**0.018**
C18:0	5.71 a	0.06	7.19 b	0.07	7.12 b	0.17	**0.024**
C20:0	0.09 b	0.00	0.11 a	0.00	0.09 b	0.00	**0.025**
C21:0	0.03 b	0.00	0.02 a	0.00	0.03 ab	0.00	**0.018**
C22:0	0.06 b	0.00	0.09 ab	0.00	0.09 a	0.01	**0.023**
C24:0	0.01 a	0.00	0.01 b	0.00	0.01 ab	0.00	**0.021**
**Σ SFA**	38.47 b	0.25	40.29 ab	0.22	42.89 a	0.47	**0.007**
C14:1	0.14	0.01	0.13	0.00	0.14	0.01	0.472
C16:1	2.86 b	0.09	2.30 a	0.04	2.31 a	0.08	**0.025**
C17:1	0.32 a	0.00	0.38 ab	0.02	0.45 b	0.03	**0.015**
C18:1n9t	0.04 a	0.00	0.04 a	0.00	0.04 a	0.00	**0.039**
C18:1n9c	25.01 b	0.13	22.71 ab	0.10	21.87 a	0.16	**0.007**
C20:1	0.26 ab	0.01	0.28 b	0.00	0.22 a	0.00	**0.021**
C22:1n9	0.02 ab	0.00	0.03 a	0.00	0.02 b	0.00	**0.010**
C24:1	0.01 b	0.00	0.01 a	0.00	0.01 ab	0.00	**0.010**
**Σ MUFA**	28.67 a	0.07	25.89 ab	0.09	25.05 b	0.10	**0.007**
C18:2n6c	28.48 ab	0.17	28.79 b	0.13	27.02 a	0.21	**0.012**
C20:2	0.19 b	0.01	0.20 ab	0.00	0.22 a	0.01	**0.023**
C22:2	0.02 b	0.00	0.02 a	0.00	0.02 ab	0.00	**0.023**
C18:3n6	0.08 ab	0.00	0.09 b	0.00	0.07 a	0.00	**0.023**
C18:3n3	2.89 b	0.01	2.79 ab	0.02	2.63 a	0.02	**0.007**
C20:3n6	0.13	0.00	0.23	0.01	0.22	0.02	**0.024**
C20:3n3	0.05	0.00	0.06	0.00	0.06	0.00	0.437
C20:4n6	0.95 b	0.02	1.54 ab	0.07	1.71 a	0.16	**0.018**
C20:5n3	0.05 b	0.00	0.06 ab	0.00	0.07 a	0.00	**0.015**
C22:6n3	0.03	0.00	0.04	0.00	0.04	0.00	0.232
**Σ PUFA**	32.86 ab	0.21	33.82 a	0.20	32.06 b	0.39	**0.012**
omega-3 (ω-3)	3.02 a	0.01	2.95 ab	0.02	2.80 b	0.01	**0.010**
omega-6 (ω-6)	29.63 b	0.20	30.65 a	0.20	29.02 b	0.39	**0.015**
(ω-6)/(ω-3)	9.807 a	0.035	10.390 a	0.120	10.366 a	0.166	**0.025**
Thrombogenicity index (TI)	0.96 b	0.01	1.04 ab	0.01	1.17 a	0.02	**0.007**
PUFA/SFA	0.85	0.03	0.84	0.02	0.75	0.04	
Atherogenicity index (AI)	0.64 b	0.01	0.66 b	0.01	0.77 a	0.03	**0.018**

a, b: Values followed by the same letter in each row do not differ significantly (*p* < 0.05, Bonferroni correction). *p*-Values in bold are statistically significant at α = 0.05.

**Table 4:** Effect of colostrum inclusion in the diet of rabbits on TVBN (mg/100 g) and TBARS (mg MDA/kg) of the meat (mean ± standard error) at 48 h postmortem (day 0) and at days 3 and 8 of shelf-life. C = control diet; BC-2.5 = diet supplemented with 2.5% bovine colostrum; BC-5 = diet supplemented with 5% bovine colostrum.

Parameter	Day	Group			Group Effect ^#^
		C	BC-2.5	BC-5	
**TVBN** **(mg/100 g)**	**0**	13.372 bA ± 0.153	15.087 aA ± 0.173	14.834 abA ± 0.183	**0.007**
	**3**	13.645 bA ± 0.281	15.212 aA ± 0.263	15.179 aA ± 0.295	**0.009**
	**8**	14.768 aB ± 0.282	15.691 aA ± 0.374	15.755 aA ± 0.516	0.146
	Time effect *	**0.005**	0.482	0.403	
**TBARS** **(mg MDA/Kg)**	**0**	0.351 aA ± 0.042	0.206 aA ± 0.011	0.192 aA ± 0.008	0.199
	**3**	0.573 aA ± 0.091	0.236 aA ± 0.085	0.274 aA ± 0.091	0.047
	**8**	0.730 aB ± 0.059	0.253 bA ± 0.077	0.295 bA ± 0.080	**0.008**
	Time effect *	**0.021**	0.910	0.721	

* Within each group. ^#^ Within each time. a, b Values followed by the same letter in each row do not differ significantly (i.e., differences between groups; *p* < 0.05; multiple comparisons using the Mann-Whitney tests). A, B Values followed by the same letter in each column do not differ significantly (i.e., differences over time; *p* < 0.05; multiple comparisons using the Mann-Whitney tests). *p*-Values in bold are statistically significant at α = 0.05.

## Data Availability

Data is contained within the article or Supplementary Material.

## Supplementary Materials

The following supporting information can be downloaded at https://www.mdpi.com/article/10.3390/foods11213433/s1: Table S1. Prevalence (number and percentage of units that are contaminated, i.e., count >1 log CFU/g) at day 0 (48 h post mortem) and during day 3 and 8 of storage for *Staphylococcus aureus*, *Enterobacteriaceae*, *E. coli*, and total coliforms. C = control diet; BC-2.5 = diet supplemented with 2.5% of bovine colostrum; BC-5 = diet supplemented with 5% of bovine colostrum.; Table S2. Numbers and percentages of unacceptable samples for each group at 48 h post mortem (Day 0), and during day 3, and 8 of storage. The following microbiological limits were set: *Staphylococcus aureus* < 4 log CFU/g, *E. coli* ≤ 4 log CFU/g, *Enterobacteriaceae* < 4 log CFU/g and total coliforms ≤ 4 log CFU/g. C = control diet; BC-2.5 = diet supplemented with 2.5% of bovine colostrum; BC-5 = diet supplemented with 5% of bovine colostrum.
