# Supplementary material for: Effect of Bovine Colostrum Dietary Supplementation on Rabbit Meat Quality
Source: Foods. 2022 Oct 29;11(21):3433. doi: 10.3390/foods11213433 (PMC9656194; doi:10.3390/foods11213433)
Supplement: Supplementary file 1 [file foods-11-03433-s001.zip › foods-1986360-supplementary.pdf]

Supplementary material

Table S1. Prevalence (number and percentage of units that are contaminated, i.e. count >1 Log CFU/g) at day 0 (48 h post mortem) and during day 3 and 8 of storage for *Staphylococcus aureus*, *Enterobacteriaceae*, *E. coli*, and total coliforms. C = control diet; BC-2.5 = diet supplemented with 2.5% of bovine colostrum; BC-5 = diet supplemented with 5% of bovine colostrum.

**Microorganism (Log CFU/g)**	**Group**	**Day 0**	**Day 3**	**Day 8**
Staphylococcus aureus	C	0_{a} (0.0%)	4_{a} (30.8%)	5_{a} (38.5%)
	BC-2.5	0_{a} (0.0%)	0_{b} (0.0%)	4_{a} (30.8%)
	BC-5	0_{a} (0.0%)	$2_{a,b}$ (15.4%)	3_{a} (23.1%)
	P value	-	0.094	0.697
Enterobacteriaceae	C	4_{a} (30.8%)	6_{a} (46.2%)	9_{a} (69.2%)
	BC-2.5	5_{a} (38.5%)	7_{a} (53.8%)	8_{a} (61.5%)
	BC-5	6_{a} (46.2%)	8_{a} (61.5%)	9_{a} (69.2%)
	P value	0.723	0.734	0.891
E. coli	C	0_{a} (0.0%)	0_{a} (0.0%)	1_{a} (7.7%)
	BC-2.5	0_{a} (0.0%)	0_{a} (0.0%)	1_{a} (7.7%)
	BC-5	0_{a} (0.0%)	0_{a} (0.0%)	1_{a} (7.7%)
	P value	-	-	1.000
Total coliforms	C	4_{a} (30.8%)	8_{a} (61.5%)	9_{a} (69.2%)
	BC-2.5	3_{a} (23.1%)	6_{a} (46.2%)	6_{a} (46.2%)
	BC-5	2_{a} (15.4%)	8_{a} (61.5%)	8_{a} (61.5%)
	P value	0.648	0.659	0.476

For each sub-table, values followed by the same letter in each column do not differ significantly ($P < 0.05$).

Table S2. Numbers and percentages of unacceptable samples for each group at 48 h post mortem (Day 0), and during day 3, and 8 of storage. The following microbiological limits were set: *Staphylococcus aureus* < 4 Log CFU/g, *E. coli* ≤ 4 Log CFU/g, *Enterobacteriaceae* < 4 Log CFU/g and total coliforms ≤ 4 Log CFU/g. C = control diet; BC-2.5 = diet supplemented with 2.5% of bovine colostrum; BC-5 = diet supplemented with 5% of bovine colostrum.

**Microorganism (Log CFU/g)**	**Group**	**Day 0**	**Day 3**	**Day 8**
Staphylococcus aureus	C	0_{a} (0.0%)	0_{a} (0.0%)	0_{a} (0.0%)
	BC-2.5	0_{a} (0.0%)	0_{a} (0.0%)	0_{a} (0.0%)
	BC-5	0_{a} (0.0%)	0_{a} (0.0%)	0_{a} (0.0%)
	P value	-	-	-
Enterobacteriaceae	C	0_{a} (0.0%)	2_{a} (15.4%)	4_{a} (30.8%)
	BC-2.5	2_{a} (15.4%)	2_{a} (15.4%)	5_{a} (38.5%)
	BC-5	0_{a} (0.0%)	5_{a} (38.5%)	7_{a} (53.8%)
	P value	0.316	0.438	0.606
E. coli	C	0_{a} (0.0%)	0_{a} (0.0%)	0_{a} (0.0%)
	BC-2.5	0_{a} (0.0%)	0_{a} (0.0%)	0_{a} (0.0%)
	BC-5	0_{a} (0.0%)	0_{a} (0.0%)	0_{a} (0.0%)
	P value	-	-	-
Total coliforms	C	0_{a} (0.0%)	4_{a} (30.8%)	6_{a} (46.2%)
	BC-2.5	0_{a} (0.0%)	3_{a} (23.1%)	3_{a} (23.1%)
	BC-5	0_{a} (0.0%)	2_{a} (15.4%)	4_{a} (30.8%)
	P value	-	0.890	0.582

a: value followed by the same letter in each row do not differ significantly ($P < 0.05$).
